# The Transfer Characteristics of Hair Cells Encoding Mechanical Stimuli in the Lateral Line of Zebrafish

**DOI:** 10.1523/JNEUROSCI.1472-18.2018

**Published:** 2019-01-02

**Authors:** Paul Pichler, Leon Lagnado

**Affiliations:** School of Life Sciences, University of Sussex, Brighton BN1 9QG, United Kingdom

**Keywords:** lateral line, mechanosensation, neuromast, ribbon synapse, zebrafish

## Abstract

Hair cells transmit mechanical information by converting deflection of the hair bundle into synaptic release of glutamate. We have investigated this process in the lateral line of larval zebrafish (male and female) to understand how stimuli are encoded within a neuromast. Using multiphoton microscopy *in vivo*, we imaged synaptic release of glutamate using the reporter iGluSnFR as well as deflections of the cupula. We found that the neuromast is composed of a functionally diverse population of hair cells. Half the hair cells signaled cupula motion in both directions from rest, either by increasing glutamate release in response to a deflection in the positive direction or by reducing release in the negative direction. The relationship between cupula deflection and glutamate release demonstrated maximum sensitivity at displacements of just ∼40 nm in the positive direction. The remaining hair cells only signaled motion in one direction and were less sensitive, extending the operating range of the neuromast beyond 1 μm. Adaptation of the synaptic output was also heterogeneous, with some hair cells generating sustained glutamate release in response to a steady deflection of the cupula and others generating transient outputs. Finally, a distinct signal encoded a return of the cupula to rest: a large and transient burst of glutamate release from hair cells unresponsive to the initial stimulus. A population of hair cells with these different sensitivities, operating ranges, and adaptive properties will allow the neuromast to encode weak stimuli while maintaining the dynamic range to signal the amplitude and duration of stronger deflections.

**SIGNIFICANCE STATEMENT** Hair cells transmit information about mechanical stimuli by converting very small deflections of their hair bundle into changes in the release of the neurotransmitter glutamate. We have measured this input/output relation in the live fish using a fluorescent protein and find that different hair cells vary in their mechanical sensitivity and the time course of their response. These variations will allow the fish to sense the timing and duration of both very weak stimuli (∼40 nm deflections) and strong stimuli (∼1 μm), underlying the ability of the fish to avoid predators and maintain its body position in flowing water.

## Introduction

An increasingly important context for the study of mechanotransduction is the lateral line of zebrafish, which is used to detect vibrations and pressure gradients in the hydrodynamic environment (Chou et al., 2017; Erickson et al., 2017; Graydon et al., 2017; Maeda et al., 2017; Oteiza et al., 2017; Sheets et al., 2017). This sensory system drives behaviors such as predator avoidance and rheotaxis- the maintenance of body position against a current (McHenry et al., 2009; Stewart et al., 2013; Olive et al., 2016). Rheotaxis operates in larval zebrafish in the absence of visual input using an algorithm that depends on the detection of flow velocity gradients on either side of the body (Oteiza et al., 2017). To understand the generation of these behaviors we therefore need to characterize how hair cells within the lateral line transfer information about mechanical stimuli.

The sense organs detecting changes in hydrodynamic pressure, the neuromasts, are distributed over the head and body, each being composed of 10–20 hair cells that project into a single cupula and therefore act as a population to signal the mechanical stimulus at that part on the body (Pujol-Martí and López-Schier, 2013). This signal is encoded as changes in the rate of glutamate release from ribbon-type synapses similar to those found in hair cells of the auditory system (Nicolson, 2015). Deflections of the cupula are encoded by a “push-pull” system in which half the hair cells are depolarized by motion in one direction and the other half by motion in the opposite direction, with segregation of these two populations onto separate afferent fibers (Faucherre et al., 2009).

Measuring the transfer characteristics of hair cells in terms of the final synaptic output is essential to understanding how mechanical information is transmitted to afferent neurons. However, a number of fundamental questions about signaling in the lateral line remain unanswered. How does glutamate release from individual hair cells encode deflections of the cupula? What is the dynamic range over which signaling occurs? And how does the output from the synaptic ribbon adapt? It is equally important to understand how these properties might vary between hair cells to determine how the population as a whole acts to encode the amplitude and duration of a stimulus.

Measuring the input-output relation of a neuromast requires assaying the release of glutamate from individual hair cells in response to measured deflections of the hair bundle. The output of hair cells has been studied by measuring capacitance changes (Beutner et al., 2001; Brandt et al., 2005; Ricci et al., 2013; Olt et al., 2014) or by recording synaptic currents in the afferent fiber (Keen and Hudspeth, 2006; Li et al., 2009; Weisz et al., 2012), but both these techniques have the disadvantage that they require the synapse to be activated by direct injection of current, bypassing the normal process of mechanotransduction. More recently, an optical approach has been used to monitor hair cell output, based on the reporter sypHy (Zhang et al., 2018). A significant drawback of sypHy, however, is that the signal it generates reflects a balance between exocytosis and endocytosis, requiring careful corrections to separate the two processes (Granseth et al., 2006; Odermatt et al., 2012). We therefore turned to the fluorescent glutamate sensor iGluSnFR (Marvin et al., 2013), which provides a more direct measure of glutamate concentrations around synaptic sites. Coupled with fluorescence measurements of cupula position, this allowed an all-optical approach to investigating the transfer characteristics of both individual hair cells and the population operating within a single neuromast.

Here we show that the output of a neuromast is determined by a heterogeneous population of hair cells that vary in mechanical sensitivity and the dynamics of adaptation, generating a population code that signals weak stimuli while maintaining the ability to encode the amplitude and duration of stronger deflections.

## Materials and Methods

### 

#### 

##### Fish husbandry.

Adult zebrafish (*Danio rerio*) were maintained in fish water at 28.5°C under a 14/10 h light/dark cycle under standard conditions (Brand et al., 2002). Fish were bred naturally and fertilized eggs were collected, washed with distilled water and transferred into 50 ml of E2 medium (concentrations in mm: 0.05 Na_2_HPO_4_, 1 MgSO_4_7H_2_O, 0.15 KH2PO_4_, 0.5 KCl, 15 NaCl, 1 CaCl_2_, 0.7 NaHCO_3_, pH 7–7.5). At 24 h postfertilization 1-phenyl2-thiourea was added to yield a final concentration of 0.2 mm to inhibit pigment formation. All procedures were in accordance with the UK Animal Act, 1986 and were approved by the Home Office and the University of Sussex Ethical Review Committee.

##### Fish lines.

The Tg[HuC::GCaMP6f] line (kindly provided by Isaac Bianco, University College London) expresses GCaMP6f pan-neuronally, including in afferent neurons of the lateral line, but not in hair cells. The Tg[Sill2, UAS::iGluSnFR] line expresses iGluSnFR (Marvin et al., 2013) exclusively in afferent neurons of the lateral line system (Pujol-Martí et al., 2012). It was generated by injecting the Sill2 construct, containing the Sill enhancer driving expression of the Gal4-VP16 element (kindly provided by Hernan Lopez-Schier, Helmholtz Zebtrum München, German Research Center for Environmental Health), into single-cell stage embryos expressing 10xUAS::iGluSnFR. Larvae were sorted for expression of iGluSnFR and reared to adulthood to identify fish with germ-line transmission (founders). To generate the Tg[Sill2, UAS::iGluSnFR, Rib::Rib-mCherry], the Sill construct was injected into the embryos derived from an outcross between 10xUAS::iGluSnFR and Rib::Rib-mCherry (Odermatt et al., 2012) adults and screened for the expression of iGluSnFR and mCherry. This line allowed us to image the iGluSnFR signal in afferent neurons while visualizing synaptic ribbons in hair cells. All fish were maintained in a nacre mutant background (Lister et al., 1999).

##### Properties of the iGluSnFR sensor.

**Figure 1.**
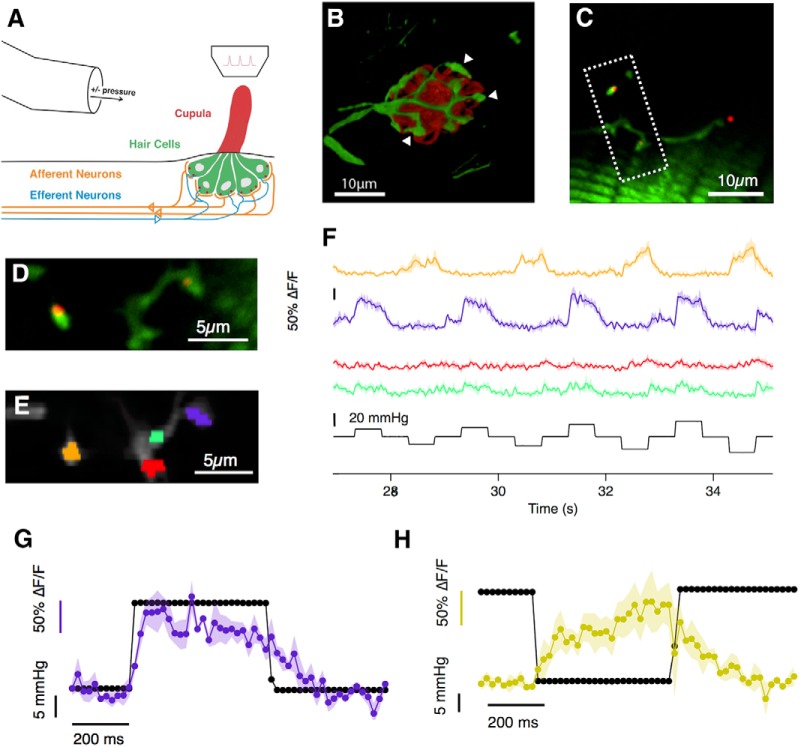
Imaging glutamate release of lateral line hair cells *in vivo*. ***A***, Neuromasts, consisting of 15–20 mechanosensory hair cells are distributed across the surface of zebrafish larvae. Upon deflection of the cupula, hair cells transmit the mechanical signal by releasing glutamate onto afferent neurons, which project into the hindbrain of the larva. Experimentally, the cupula of a neuromast of the posterior lateral line (L3–L6) was stimulated with positive and negative pressure steps applied through a bent pipette along the anterior–posterior direction of the larva. The glutamate released by hair cells was imaged through a two-photon microscope. ***B***, The fluorescent glutamate reporter iGluSnFR was expressed over the surface of afferent neurons (green), which form a basket-like structure around hair cells, here counterstained in red using FM4-64. White arrowheads indicate such postsynaptic varicosities. ***C***, Image of a neuromast in a larva at 7 dpf [Tg(Sill2, UAS::iGluSnFR, Rib::Rib-mCherry)] showing varicosities of one afferent neuron (green) as well as synaptic ribbons at the basal side of hair cells (red). ***D***, Close-up of the boxed area in ***C***. Of the four visible varicosities, two did not coincide with presynaptic ribbons. ***E***, ROIs used for analysis. ***F***, The responses of the four ROIs in ***E***. The amber and purple ROIs, postsynaptic to ribbons, responded to positive and negative pressure steps, respectively. The red and green ROIs not opposed to ribbons did not respond. (Shaded area indicates SEM of 3 repeats.) ***G***, ***H***, Magnification of the responses to a single positive and negative pressure step of the purple and amber ROIs, respectively. Markers are 20 ms apart (image sequence was acquired at 50 Hz) and an initial peak of the response is reached within 100–150 ms.

We used the iGluSnFR variant developed by Marvin et al. (2013), which is maximally excited at ∼930 nm in the two-photon regime and has a dynamic range of Δ*F*/*F* = 4.5. This variant responds rapidly to synaptic release of glutamate: transients generated by one vesicle reach a peak within ∼10 ms and then decay with a time constant of 30–50 ms, as measured in hippocampal cultures (Marvin et al., 2013) and *in vivo* at ribbon synapses in the retina of larval zebrafish (Lagnado et al., 2018). The response of iGluSnFR also appears linear over a wide range: in retinal ganglion cells, the change in iGluSnFR fluorescence is directly proportional to the glutamate activated current across at least a 20-fold range of visually evoked responses (Borghuis et al., 2013). These properties indicate that the detection of fast changes in synaptic glutamate concentration was limited by the sampling frequency of the experiments rather than the off-rate of the reporter. In a typical experiment imaging at 10–50 Hz we were able to resolve the decline in the iGluSnFR signal at the end of a large step displacement (recovery to baseline within ∼250–400 ms; Fig. 1*F*), as well as the time constants of adaptation (averaging 600 ms; Fig. 7).

##### Mounting and cupula staining.

**Figure 2.**
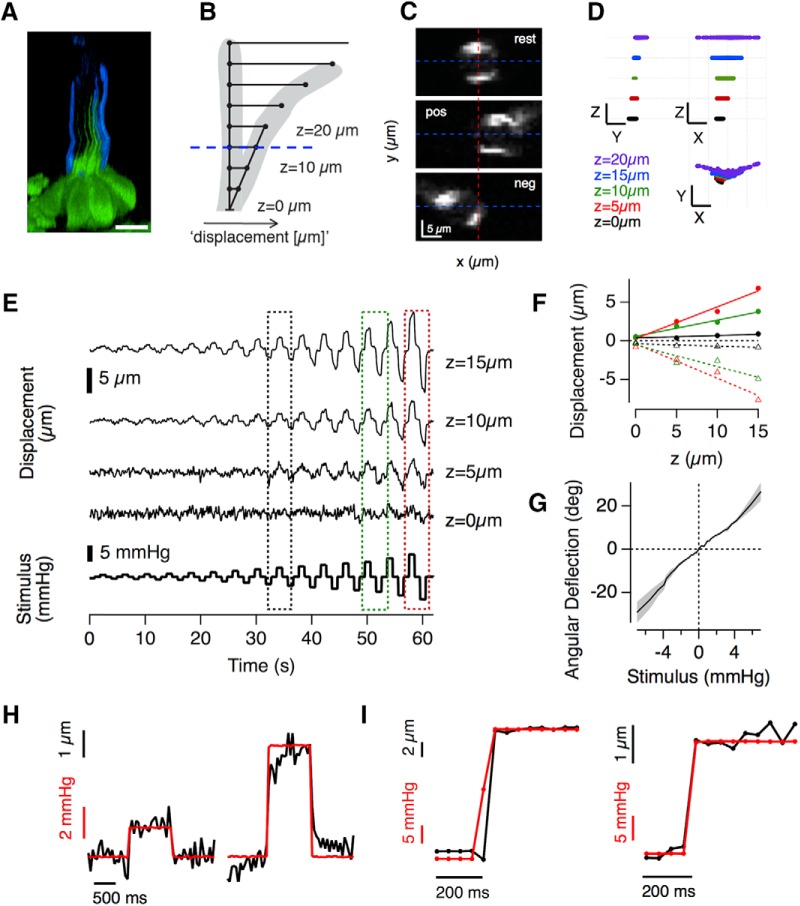
Optically assessing cupula deflection–the mechanical input of lateral line hair cells. ***A***, Side projection of a neuromast expressing GFP in hair cells with the surface of the cupula stained with AlexaFluro-350-WGA. Notice how the kinocilia extend approximately half way up the cupula. The staining was not evenly distributed across the surface of the cupula. Scale bar, 10 μm. ***B***, Schematic of the model used to calculate angular deflections of the cupula from a pivot at its base. The translational deflection for a given stimulus pressure was measured at several distances *z* above the apical surface of the hair cell. ***C***, Three representative frames of the stained cupula at *z* = 15 μm (***B***, blue dashed line) at rest and deflected by a positive (pos) and negative (neg) pressure step. The angular deflection was calculated as tan^−1^ (Δ*x*/*z*), where Δ*x* was the translation in the center of mass of the staining from the rest position (dashed red line indicated center position). ***D***, Cupula motion in space over the course of the entire experiment can be extracted from these data. Stimulation along the *x*-axis (parallel to the fish), led to increasingly strong translational displacements toward the tip of the cupula along the *x*-axis and hardly induced motion along the orthogonal, *y*-axis. Scale bars: *x*, *y*, 10 μm; *z*, 5 μm. ***E***, Traces showing *x* displacement as a function of time at four different *z* distances and for a variety of pressure steps (bottom trace). The image sequences were obtained at 20 Hz. ***F***, The *x* displacements to positive and negative pressure steps of increasing magnitude (black, green, and red, corresponding to stimuli delivered in boxes shown in ***E***). Note that *x* displacement is directly proportional to *z* at any pressure, indicating that the proximal part of the cupula indeed acts as a beam, deflecting at a pivot point at its base. Several measurements within this rigid region of the cupula could therefore be averaged. ***G***, Example of a calibration curve relating the stimulus pressure to the angular deflection (average of several Δ*x* measurements in the “rigid” part of the cupula), gray shading indicated SEM, see Materials and Methods. These relations were generally linear. ***H***, Translational displacement (same as in ***F***) of the cupula at *z* = 10 μm for an intermediate (left) and strong (right) deflection step. Although the intermediate pressure step led to a deflection within 1 sample point (50 ms), an additional slow small creep was apparent in some of the stronger steps. ***I***, Two examples of translational displacements of cupulae at *z* = 10 μm to saturating pressure steps in which the applied pressure led to a steady deflection within 1 sample point (50 ms, indicated by markers).

All experiments were performed at room temperature (20–25°C) using larvae at 7–10 d postfertilization (dpf). At 4 dpf, embryos were screened for the strongest expression of the respective transgene. Larvae were anesthetized by immersion in 0.016% tricaine (MS-222), diluted in E2. They were then placed side-down into a “fish-shaped” pit carved into a thin (∼1 mm) layer of PDMS (Sylgard184, Dow Crowning) on a coverslip. Mechanical stability was provided by a 'harp' (Warner Instruments) placed on top of the larva. The pressure applied by the nylon strings was adjusted to allow normal blood flow while maintaining enough pressure to hold down the larva. The larva was then paralyzed by injection of 0.25 mm α-Bungarotoxin (Tocris Bioscience) into the heart. To avoid damaging the cupula, special care was taken not to touch the upward-facing side of the fish during the mounting procedure. The cupula was then stained by incubating the fish in a 1:500 dilution of 1 mg/ml WGA AlexaFluor-594 or WGA AlexaFluor-350 (Life Technologies) for 2 min followed by thorough washing with E2. This staining was not always evenly distributed across the surface of the cupula (Fig. 2*A*,*C*). When counter-staining hair cells with FM4-64 (Synaptored, Biotium) larvae were incubated in a 1 μm solution for 1 min and then washed with E2.

##### Two-photon imaging.

Fish of either sex were imaged on a custom built two-photon microscope driven by a mode-locked Titanium-sapphire laser (Chameleon 2, Coherent) tuned to 915 nm (Odermatt et al., 2012). Excitation was delivered through a 40× water-immersion objective (Olympus, 40× LUMIPlanF, NA: 0.8) and emitted photons were collected both through the objective and an oil condenser (NA 1.4, Olympus) below the sample. Visible emission was separated from IR light by a dichroic mirror (760dcxru) above the objective and focused onto a GaAsP photodetectors (H10770PA-40, Hamamatsu). A filter slider in front of the detector was used to switch between green (525/70 nm) and red (620/60 nm) emission filters. A second detector below the condenser only collected green emission through a 530/60 nm filter. Dual color stacks of Tg[Sill2, UAS::iGluSnFR, Rib::Rib-mCherry] were acquired by simultaneously exciting the iGluSnFR and mCherry at 1030 nm and collecting the emitted photons through the objective (red 620/60 nm emission filter) and condenser (green 530/60 nm emission filter), respectively. Photocurrents generated by the detectors were passed through a transimpedance amplifier (Model SR570, Stanford Research Systems) and low-pass filtered (300 kHz). When only the iGluSnFR signal was to be recorded, the currents from both photodetectors were summed before the amplification step to increase the signal-to-noise ratio. The microscope was controlled by ScanImage v3.8 (Vidrio Technologies) and image acquisition was synchronized with the stimulus. Image sequences were acquired at 10–50 Hz.

##### Mechanical stimulation.

Pressure steps were applied to a neuromast through a glass pipette attached to a high speed pressure clamp (HSPC-1, ALA Scientific; Trapani et al., 2009). The output pressure (as measured at the back of the pipette) was controlled using mafPC software (courtesy of M. A. Xu-Friedman, University of Buffalo) running in IgorPro (WaveMetrics) and synchronized to image acquisition. (The applied pressure as measured at the end of the pipette was recorded as a separate channel in ScanImage.) The micropipette was pulled to a diameter of ∼30 μm and the tip bent through 30° using a micro forge (Narishige) to allow liquid flow parallel to the body of the larva. The tip was positioned ∼20 μm above the body, ∼100 μm from the neuromast. Before approaching the neuromast the pressure clamp offset was set so that the sensor monitoring the pressure at the back of the pipette was at zero and produced no net flow. This study was confined to neuromasts of the posterior lateral line (L3–L6) with an “anterior–posterior” axis of sensitivity. The direction of the pipette (pointing toward the tail or toward the head) was changed during the course of some experiments but did not affect measurements.

##### Measuring deflections of the cupula.

The angular deflection of the stained cupula was assessed by measuring its translational displacement in multiphoton images in planes at different *z* distances from the surface of the hair cells. The measurements were made at a variety of stimulus pressures and repeated in 3–4 planes in 5 μm increments. The central position of the cupula within each frame was extracted by first thresholding the image and then fitting an ellipse to estimate the center of mass. Next, the translational deflections induced by the applied pressure steps in each plane were calculated: these were consistent with the proximal regions of the cupula (*z* < 15–20 μm) behaving as a pivoting beam (McHenry and van Netten, 2007). The angular deflection was then calculated as tan^−1^ (Δ*x*/*z*), where Δ*x* was the translation within the plane and *z* the height above the surface of the hair cell. This angle was calculated in each of the planes and subsequently averaged. In this way, a calibration of the angular deflection of the cupula for each stimulus pressure was obtained for each experiment. This relationship was linear, and the slope is indicated by the degree/millimeter mercury calibration in the respective figure legends. This calibration was repeated if, for instance, the pipette was moved.

The pressure steps used to stimulate the cupula were filtered by the mechanical properties of the pressure clamp, the hydrodynamics of the fluid and the mechanical properties of the cupula itself. Under the experimental conditions mentioned above (pipette diameter of ∼30 μm, ∼100 μm away from the cupula) and for weak to intermediate pressure steps, this meant that the cupula usually reached its “final” deflection within 50 ms after stimulus onset (one sample point in Fig. 2*H*, left, *I*). For stronger stimuli, the initial, fast deflection was sometimes followed by a slow creep (Fig. 2*H*, right).

##### Imaging protocol.

After the larva was placed under the two-photon microscope an afferent neuron expressing iGluSnFR and innervating an appropriate neuromast (L3–L6) was identified. Subsequently the stimulating pipette, connected to the zeroed HSPC1 was manually approached while monitoring the cupula position through the eyepieces of the microscope to ensure that the resting pressure of the pressure clamp did not alter the cupula's resting position. After the pipette was positioned, a high-resolution 3D image stack was taken for later reference [a dual color stack in the case of the Tg(Sill2, UAS::iGluSnFR, Rib::Rib-mCherry)]. The focal plane was chosen to capture a good number of varicosities in labeled afferent neurons, indicating the positions of hair cell synapses. We imaged an average of 5.1 active varicosities per neuromast.

The dynamic range of the set of varicosities within the focal plane was initially assessed coarsely using a stimulus protocol consisting of three positive and three negative pressure steps of varying amplitude. After that, a longer stimulus protocol was applied involving smaller increments in pressure (usually 15–20 positive and negative) allowing a more detailed construction of the mechanical tuning curve. To allow averaging, this protocol was repeated three times with ∼20–30 s breaks in between. If the response of a synapse declined significantly during these three repeats it was discarded from the analysis. Such changes could occur through photobleaching of the iGluSnFR protein reducing the SNR, the pipette being clogged by debris or drifting motion of the preparation. After delivery of stimulus protocols and imaging of iGluSnFR responses, the pressure-deflection relationship was established by using a protocol consisting of the same positive and negative pressure steps (described above).

##### Image analysis and statistics.

Images were analyzed in Igor Pro (Wavemetrics) using custom-written software including the SARFIA toolbox (Dorostkar et al., 2010). Image sequences containing small drifts in the *x–y* dimension were registered but those with large drifts, including potential *z*-motions, were discarded. Regions-of-interest (ROIs) were determined using an algorithm that began by identifying pixels with both large signals and high degrees of temporal correlation. Pixels surrounding thee ROI “seeds” were added to the ROI until the correlation value fell below a threshold. Background fluorescence was subtracted by manually choosing a region within the image sequences that did not contain a stimulus-dependent signal and subtracting the average in that background ROI from the average in the varicosity ROI on a frame by frame basis. Baseline fluorescence (F) was defined as the average fluorescence preceding the first stimulation interval. The change in fluorescence relative to baseline (Δ*F*/*F*) was calculated and used for further analysis. Every response constitutes the average of the three stimulus repetitions.

The population data in Figure 4 were generated as follows: for each ROI, signals were measured for a wide range of cupula deflections, yielding ∼2800 paired measurements of Δ*F*/*F* and cupula deflection, from 67 hair cells in 13 fish. The measurements were accumulated by sorting these signals according to the deflection absolute angle of the cupula (pooled positive and negative deflections), binning them and then averaging responses within the bin.

The adaptation index (AI) was calculated as follows:

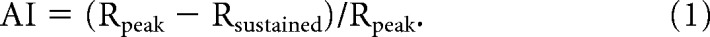
 Where *R*_peak_ is the instantaneous peak response after stimulus onset and *R*_sustained_ is the average responses during the last 100 ms of stimulus presentation. Therefore, an AI close to 0 indicates no adaptation, an AI close to 1 indicates complete adaptation and a negative AI indicates sensitization.

The relative set-point (SPr) was calculated as follows:

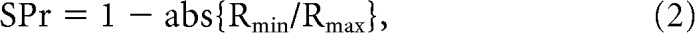
 where *R*_max_ is the saturating response in the preferred direction of deflection and *R*_min_ is the maximum change in the opposite direction.

Experimental values in the text and results on graphs are expressed as mean ± SEM. Errors estimated for parameters fitting functions to results are expressed as ± SD. Potential correlations between the half-angles, working ranges (WRs), and relative set-points measured within individual neuromasts were investigated using the Spearman rank correlation test.

Leon Lagnado should be contacted for any data or material requests.

## Results

### An all-optical approach to measuring the transfer characteristics of hair cell ribbon synapses *in viv*o

The lateral line system has been studied intensively but the input/output relation of hair cells within neuromasts is still unclear. To observe the output in larval zebrafish we used the Sill promoter to drive expression of the fluorescent glutamate sensor iGluSnFR in the surface membrane of primary afferents postsynaptic to hair cell ribbons (Pujol-Martí et al., 2012; Marvin et al., 2013; Fig. 1*A–C*). Neuromasts in the posterior lateral line were stimulated using a narrow pipette that applied positive and negative pressure steps, generating iGluSnFR signals at distinct hotspots (Fig. 1*D–F*). These hotspots were identified as the outputs of ribbon synapses based on two pieces of evidence. First, the hotspots coincided with varicosities of the afferent fiber, recognizable by their bulged morphology (Fig. 1*B*, white arrowheads), which have previously been shown to constitute stable afferent synapses onto single hair cells (Faucherre et al., 2009). Second, iGliSnFR hotspots coincided with presynaptic ribbons labeled using Ribeye-mCherry (Odermatt et al., 2012). Figures 1,*D* and *E*, home in on four varicosities from two afferents: two of the ROIs were contacted by one ribbon (amber and purple ROIs), whereas the other two were not (red and green ROIs). iGluSnFR signals were only observed in areas that were in close apposition to a ribbon synapse (Fig. 1*F*), and this was the rule in all three neuromasts in which this test was made.

To quantify the mechanical input to the hair cells, deflections of the cupula were visualized by staining polysaccharides on the surface blue or red with AlexaFluor-350/594 coupled Wheat Germ Agglutinin (Fig. 2*A*,*C*; detailed in Materials and Methods). The rotational deflection of the cupula was calculated based on the idea that the proximal region acts as a rigid lever pivoting on a plane at the apical surface of the hair cells (Fig. 2*B*; McHenry and van Netten, 2007). We tested this model by imposing a variety of pressure steps (Fig. 2*E*, bottom) and tracking the translational motion of the cupula through planes at four different distances from the apical surface of the hair cell. Figure 2*C* shows images of the cupula at *z* = 15 μm, from which the *x* translation was estimated from the movement of the center of mass of the fluorescence. At any single pressure, the *x* translations at different *z* gave consistent estimates of the angle of rotation confirming that the lower part of the cupula behaves as a beam pivoting at its base (Fig. 2*D*,*F*). We were therefore able to calibrate the relation between applied pressure and rotation of the cupula for each experiment (Fig. 2*G*). This calibration was necessary because of variations in experimental factors such as the diameter and angle of the pipette delivering the stimulus, as well as biological factors altering the flexural stiffness of the cupula such as its height and the number of kinocilia embedded within it (McHenry and van Netten, 2007).

The proximal region of the cupula contains the kinocilia and hair bundles of all the hair cells within the neuromast (Fig. 2*A*), so the angle of the cupula quantifies the sensory input to the neuromast organ as a whole. Crucially, we were able to relate this stimulus to the output from multiple hair cells within a neuromast. For example, Figure 1*F* demonstrates the simultaneous measurement of activity from synapses of opposite polarity: whereas the purple ROI was activated by positive deflections toward the head, the amber ROI was activated by negative deflections toward the tail.

It is important to consider the temporal relation between the command signal delivered to the device controlling the stimulation pipette, the deflection of the cupula and the iGluSnFR signal recorded. In the majority of experiments the deflection of the cupula reached a steady value within one or two sample points, equivalent to 50–100 ms (Fig. 2*H*,*I*), regardless of applied pressure. In some cases, however, the strongest pressure steps generating saturating iGluSnFR signals also caused a slower creep in cupula deflection (Fig. 2*H*, right), equivalent to ∼15% of the initial displacement. The source of this creep was not clear and in these cases the stimulus applied to the neuromast was quantified as the initial fast deflection.

The iGluSnFR signal indicating an increase in glutamate release appeared slightly delayed relative to cupula deflection, reaching an initial peak 100–150 ms from the onset of the command signal, as shown in Figure 1, *G* and *H*. These kinetics are not limited by the reporter itself: glutamate released from a single vesicle generates an iGluSnFR signal that peaks within 5–10 ms (Marvin et al., 2013; Helassa et al., 2018; Lagnado et al., 2018). The off-rate of the iGluSnFR variant we used is also relatively fast, the transient generated by a single vesicle decaying with a time-constant of 15–50 ms. The decline in iGluSnFR signals at the end of a pressure step was significantly slower (Fig. 1*G*,*H*), indicating that it was rate-limited by factors such as the cessation of glutamate release or the clearance of the transmitter rather than by dissociation of glutamate from iGluSnFR. The ability to resolve changes in synaptic glutamate concentration was therefore limited by the sampling frequency in our experiments rather than the kinetics of the sensor (Materials and Methods).

### Ribbon synapses in the lateral line can signal deflections <100 nm

What is the mechanical sensitivity of hair cells in the lateral line? Relating spikes in the afferent nerve to estimates of cupula deflection, Haehnel-Taguchi et al. (2014) report that deflections <8 μm cannot be encoded. In contrast, calcium imaging in the hair cells themselves indicate that calcium responses can be elicited by deflections between ∼1 and 3 μm (Kindt et al., 2012; Sheets et al., 2012; Zhang et al., 2016). It is less clear how these calcium signals engage the exocytotic machinery and a recent report suggests that the efficiency with which calcium triggers release might vary between different ribbon synapses in the lateral line (Olt et al., 2014). Imaging glutamate release using iGluSnFR provided a more direct assay of the final output of hair cells in the lateral line.

**Figure 3.**
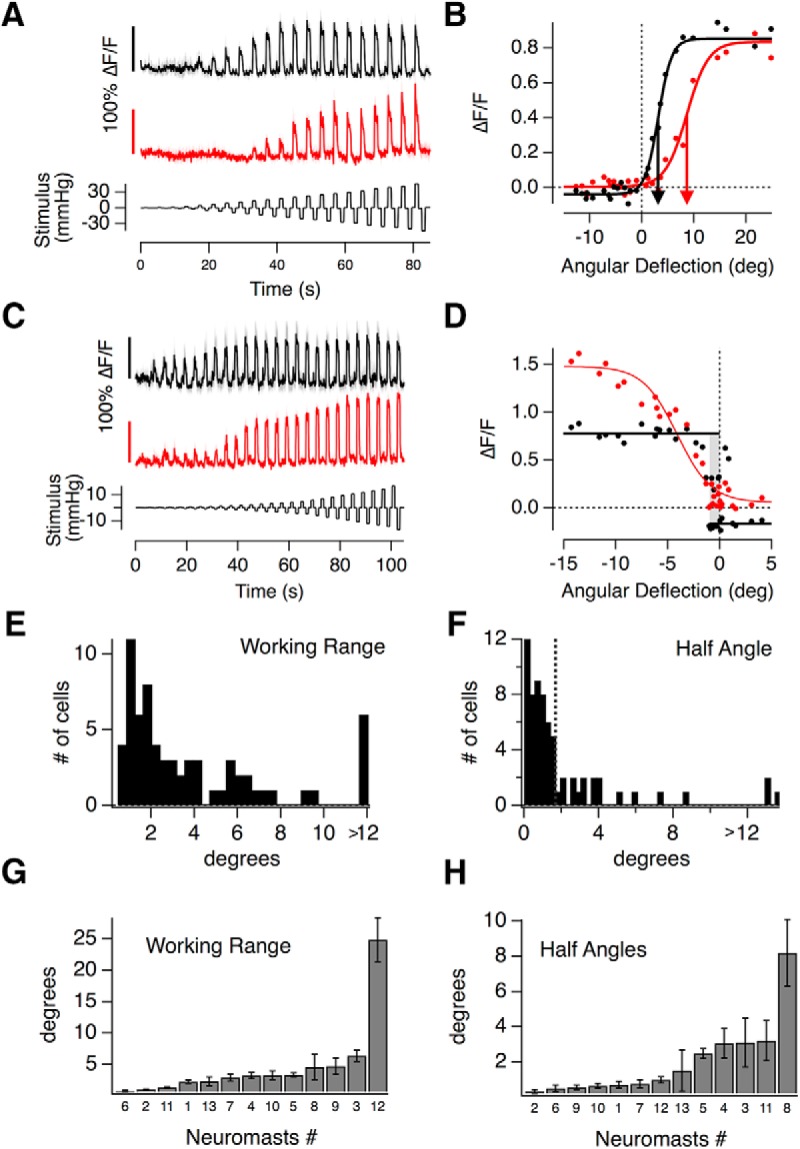
The transfer characteristics of hair cells in the lateral line. ***A***, iGluSnFR responses of two hair cells recorded in the same neuromast, both responding to positive pressure steps. The top one (black) responds to smaller deflections than the bottom one (red). The relationship between cupula deflection and applied pressure in this neuromast was 0.6 deg/mmHg. ***B***, Peak iGluSnFR signals (*R*) from ***A*** plotted as a function of the angular deflection of the cupula (*X*). These stimulus-response relations could be described by a two-state Boltzmann function (Eq. 3), with parameters *R*_max_, *R*_min_, *X*_1/2_, and *X*_s_: *R*_max(1)_ = 0.89 ± 0.02, *R*_min(1)_ = −0.04 ± 0.01, *X*_1/2(1)_ = 3.19 ± 0.15°, and *X*_s(1)_ = 1.28 ± 0.15, *R*_max(2)_ = 0.83 ± 0.02, *R*_min(2)_ = 0.00 ± 0.01, *X*_1/2(2)_ = 8.65 ± 0.34°, and *X*_s(2)_ = 2.07 ± 0.3. ***C***, iGluSnFR responses of two hair cells from another neuromast, which differ more significantly in their WR. ***D***, Stimulus–response relations of the hair cell in ***C***. The hair cell depicted in red had a WR of 7°, and the one in black <1° (gray bar). ***C***, ***D***, The relationship between applied pressure and measured cupula deflection in this neuromast was 1.9 deg/mmHg. ***E***, A histogram of WRs (the deflection required to increase the response from 10 to 90% of maximum) measured in 67 hair cells. Approximately 30% have WRs within 1.5°. The last bin contains all hair cells >12°. ***F***, Histogram of the half angles (*X*_1/2_, the rotation at half maximum response) in 67 hair cells. The majority (75%) had *X*_1/2_ < 2° (dashed line). ***G***, ***H***, The average WRs and half-angles of hair cells from 13 different neuromasts, ranked in order. The neuromast number is indicated below each bar. No correlation between these metrics could be detected using a Spearman rank correlation test (error bars indicate SEM).

An experiment measuring the input/output relation of two nearby hair cells is shown in Figure 3*A*: iGluSnFR signals were measured in response to positive and negative pressure steps of increasing amplitude, each lasting 1 s. It can be immediately seen that hair cell 1 (black trace) was more sensitive to small deflections from rest, generating changes in glutamate release at stimulus strengths that did not elicit glutamate release from hair cell 2 (red trace). The mechanical tuning of these receptors was characterized as the peak amplitude of the iGluSnFR signal (R) as a function of angular rotation of the cupula (X), as plotted in Figure 3*B*. A good empirical description of this relation was provided by a Boltzmann equation of the form:

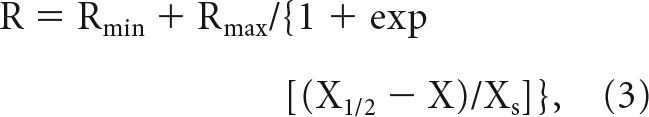
 where *R*_max_ is the saturating response in the preferred direction, *R*_min_ is the maximum change in the null direction, *X*_1/2_ is the rotation that half-activates (half angle), and *X*_S_ is the slope factor.

The sensitivity of a sensory system can be quantified as the change in response per unit change in stimulus (Dayan and Abbott, 2001; Butts and Goldman, 2006). In Equation 3, *X*_1/2_ is the point at which the gradient of the function is steepest, and therefore defines the deflection at which the sensitivity of the hair cell is at its maximum (Dayan and Abbott, 2001; Butts and Goldman, 2006). The two synapses in Figure 3*B* differed significantly in *X*_1/2_ (arrowed) and a survey of half-angles across 67 hair cells from a total of 13 neuromasts is shown by the histogram in Figure 3*F*. Approximately 70% of synapses displayed half-angles <2°; assuming that hair bundles move in tandem with the cupula, this corresponds to deflections <170 nm at the top of the hair bundle ∼5 μm tall (McHenry et al., 2008; Maeda et al., 2017). We conclude that the majority of hair cells in the lateral line have a mechanical sensitivity comparable to auditory hair cells in mice and other species (for review, see Fettiplace and Kim, 2014).

The variability in the average WR and half-angle measured within individual neuromasts is shown in Figure 3, *G* and *H*. Between 2 and 10 hair cells were sampled in each (average 5), which constitutes 10–50% of the total. There was no significant correlation between the average WR of the hair cells sampled within a neuromast and their average half-angle (Spearman rank correlation test). The variability in the averaged properties is likely to reflect the functionally heterogeneous populations of hair cells within each neuromast.

### Heterogeneous transfer characteristics of hair cells within individual neuromasts

Hair cells within neuromasts also varied significantly in their WR- the deflection required to increase the response from 10% to 90% of maximum (Markin and Hudspeth, 1995). The hair cells featured in Figure 3, *A* and *B*, operated over relatively broad WRs of 490 nm (5.6°) and 790 nm (9.0°), respectively. Other hair cells, however, operated with much narrower WRs of 90 nm (∼1°), as shown by the black trace in Figure 3*C* and corresponding plot of the transfer function in Figure 3*D*. The coexistence of hair cells with significantly different transfer characteristics was again evident in this neuromast, where another cell signaled deflections with a WR of 630 nm (Fig. 3*C*, *D*, red traces).

The distribution of WRs across 67 hair cells from 13 neuromasts is shown by the histogram in Figure 3*E*, which displayed an initial peak followed by a long tail. The peak contained ∼60% of hair cells and was centered at ∼1.5°, which is equivalent to deflections of 130 nm. These estimates fall within the range of measurements of the WR made in auditory and vestibular hair cells of a number of species (20–400 nm; Fettiplace and Kim, 2014). The other 40% of cells signaled much larger deflections, with WRs between 5° and 30° (0.5–2.5 μm).

**Figure 4.**
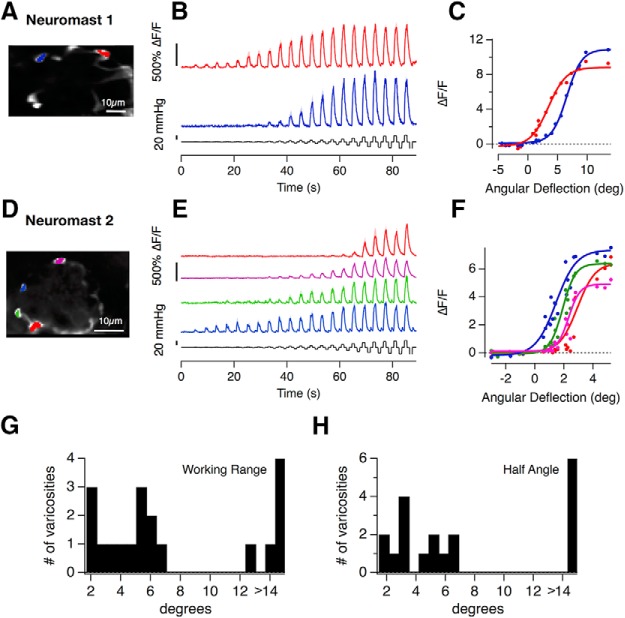
Heterogeneous transfer characteristics of hair cells revealed by measuring calcium signals in afferent neurons. ***A***, ***D***, The Tg[HuC::GCaMP6f] line expresses the calcium indicator GCaMP6f in afferent neurons of the posterior lateral line but not in hair cells. ***A–F***, The variety of stimulus response relations from postsynaptic varicosities in two neuromasts. In Neuromast 1 the red and blue traces in (***B***, ***C***) have an *X*_1/2_ of 3.5° and 6.6° and a WR of 6.6° and 5.5°, respectively. In Neuromast 2 (***D***–***F***) *X*_1/2_ ranges from 1.4° to 3° and the WR spans from 1.7° to 3°. The relationship between cupula deflection and applied pressure was 0.74 deg/mmHg in Neuromast 1 and 0.26 deg/mmHg in Neuromast 2. ***G***, ***H***, The distribution of WRs and half-angles in 19 postsynaptic varicosities from *n* = 4 neuromasts in four fish.

Are these variations in the transfer characteristics of hair cells also evident in the activity of postsynaptic afferents? To investigate this question, imaged calcium signals in the afferents using GCaMP6f under the pan-neuronal *HuC* (*elavl3*) promoter (Fig. 4). These experiments revealed that the half-angles and WRs of afferent responses also varied significantly between different synaptic contacts in the same neuromast (Fig. 4*A–F*), as well as in the population of neuromasts (Fig. 4*G*,*H*).

A number of processes between the mechanotransducer channel and the ribbon synapse might contribute to these variations in overall transfer characteristics of hair cells. For example, the input resistance of hair cells might vary such that a given MET current causes variable levels of depolarization; the voltage dependence of L-type calcium channels might alter depending on the action of neuromodulators, or the calcium dependence of the release process might change. Indeed, developmental heterogeneity of hair cells has been identified by measuring their electrophysiological properties (Olt et al., 2014) and calcium responses (Kindt et al., 2012). Furthermore, the existence of hair cells with different levels of functional maturity continues beyond the larval stage into the adult fish because hair cells undergo continuous turnover (Cruz et al., 2015). Whatever the underlying mechanisms, these results demonstrate that the neuromast encodes deflections of the cupula through a mixed population of receptors operating over different ranges. These heterogeneous transfer characteristics will likely determine the performance of the lateral line system in driving behaviors such as predator avoidance and rheotaxis as they are studied in these larval animals (Stewart et al., 2013; Oteiza et al., 2017).

### Individual hair cells can encode opposing directions of motion

**Figure 5.**
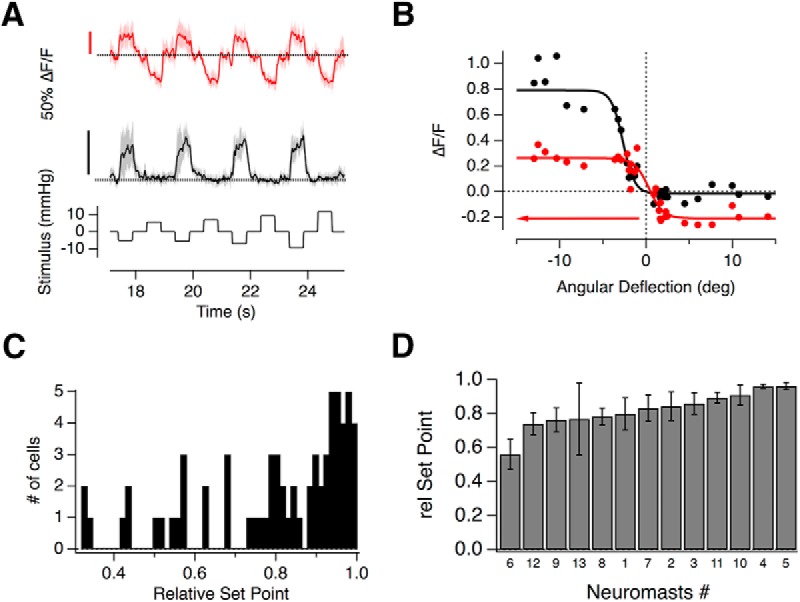
Push-pull signaling in individual hair cells. ***A***, The glutamate release from two hair cells in the same neuromast was measured using the iGluSnFR probe. Although both hair cells were polarized in the negative direction, the one depicted in black was completely rectifying but the hair cell in red could also clearly signal deflections in the positive direction as a decrease in glutamate release. The relationship between cupula deflection and applied pressure in this neuromast was 0.7 deg/mmHg. ***B***, Stimulus–response relations of the hair cells in ***A***. *R*_max(1)_ = 0.81 ± 0.04, *R*_min(1)_ = −0.02 ± 0.07, *X*_1/2(1)_ = −2.68 ± 0.18°, and *X*_s(1)_ = 0.67 ± 0.16, *R*_max(2)_ = 0.47 ± 0.03, *R*_min(2)_ = −0.21 ± 0.05, *X*_1/2(2)_ = 0.32 ± 0.36°, and *X*_s(2)_ = 0.83 ± 0.22. (Axis is reversed to represent the sensitivity to negative deflections.) The relative set-points of the black and red relations were 1 and 0.4, respectively. ***C***, Distribution of the relative set-points from 67 hair cells. Although the majority were strongly rectifying with relative set-points close to 1, there was a large degree of variability. ***D***, The average relative set-points of hair cells within the 13 neuromasts in increasing order.

The two populations of hair cells polarized to opposite directions allow the neuromast to encode the direction of a stimulus using a push-pull system, similar in principle to the ON and OFF channels in the retina (Ghysen and Dambly-Chaudière, 2007; Masland, 2012). We found, however, that hair cells varied greatly in their ability to encode opposing directions of motion. For example, Figure 5*A*, red trace, shows a synapse where deflections of the cupula in the nonpreferred direction caused a large decrease in glutamate release: the input/output relation from this hair cell shows that 40% of its dynamic range was used to signal motion in the null direction (Fig. 5*B*). In contrast, another hair cell in the same neuromast was completely rectifying, only modulating release in the preferred direction (Fig. 5*A*,*B*, black trace).

The ability of individual hair cells to signal opposite directions of motion was quantified as the “relative set-point” for glutamate release- the fraction of the total dynamic range of the synapse modulated by deflections from rest in the preferred direction (Fettiplace and Kim, 2014). A synapse in which the maximum amplitude of the change in the iGluSnFR signal was equal for the positive and negative directions would, for instance, have a relative set-point of 0.5 (see Materials and Methods; Eq. 2). The distribution of relative set-points across a sample of 67 hair cells is shown by the histogram in Figure 5*C*. In 27% of synapses this fraction was <0.7, i.e., >30% of the dynamic range was used to signal deflections in the null direction as a decrease in the rate of glutamate release. The variability in the relative set-point averaged within individual neuromasts is shown in Figure 5*D*. There was no significant correlation between this property and either the WR or half-angle measured within the 13 neuromasts (Spearman rank correlation test).

These results demonstrate that although the output from most hair cells in the posterior lateral line rectify strongly, the large majority can encode deflections of the cupula both toward and away from the head. This property will allow for larger differential signals in the two afferents, whereby an increase in the spike rate of one occurs simultaneously with a decrease in the rate of the second to below the spontaneous rate in the absence of a stimulus.

### A mixed population of high- and low-sensitivity hair cells

**Figure 6.**
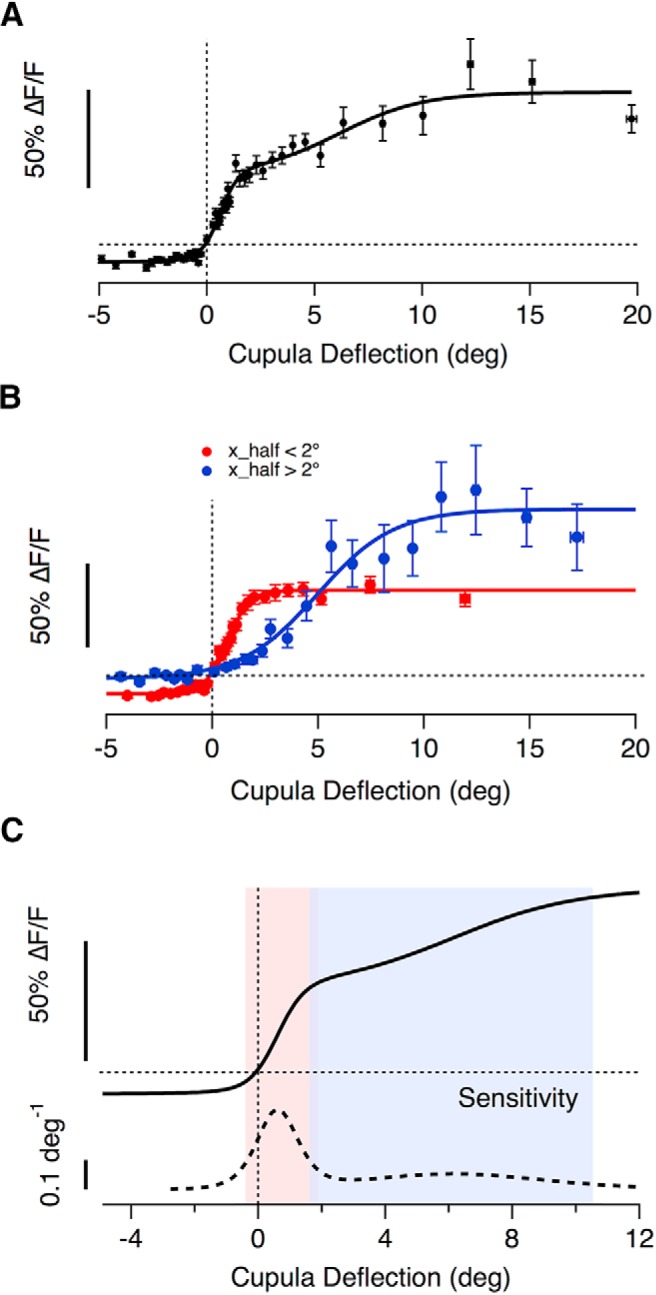
The average transfer characteristics of hair cells in a neuromast. ***A***, The binned averages of 2800 individual paired measurements of cupula deflection and peak glutamate release, recorded from 67 hair cells using the iGluSnFR probe. All responses are plotted as a function of the magnitude of deflection, regardless of direction. A good empirical description was provided by the sum of two Boltzmann relations (Eq. 3) as shown by the fitted curve (*R*_min_ = 0.1 ± 0.02, *R*_max(1)_ = 0.44 ± 0.09, *R*_max(2)_ = 0.42 ± 0.12, *X*_1/2(1)_ = 0.60 ± 0.1°, *X*_1/2(2)_ = 6.14 ± 1.17°, *X*_s(1)_ = 0.42 ± 0.12, *X*_s(2)_ = 1.95 ± 0.95). The WR of the whole population is 8.9°. ***B***, The average stimulus–response relation of hair cells separated into two groups based on half-angle with a threshold of 2°. The two subsets had average half-angles of 0.7° (red, *n* = 50 hair cells) and 5° (blue, *n* = 17 hair cells). The high- and low-sensitvity groups of hair cells also differed significantly in *R*_min_, the maximum change in the null direction, with values of −0.11 ± 0.01 and −0.02 ± 0.02, respectively. The working rages of these populations were 2.3° and 8° respectively and overlapped between 1° and 1.9°. ***C***, The sensitivity of the whole population calculated as the derivative of the fit in ***A***. Small deflections (<2°) are encoded with high sensitivity by the large population of hair cells whereas the second smaller population extends the dynamic range significantly to also capture larger cupula deflections, ranging beyond 10°.

How do hair cells with these different transfer characteristics act as a population to encode a deflection of the cupula? To obtain an overall picture of how the neuromast operates we estimated the total input to a single afferent by averaging the stimulus-response relation from 67 hair cells, assuming that hair cells of opposite polarity were, on average, mirror-images of each other (Fig. 6*A*). This assumption was based on the observation that the stimulus-response relations of hair cells signaling deflections toward the head were not significantly different from those signaling deflections toward the tail. The tuning curve averaged over all hair cells could be described as the sum of two sigmoid functions with significantly different slope factors and half-angles (*X*_S(1)_ = 0.4 ± 0.1, *X*_S(2)_ = 1.9 ± 0.9, *X*_1/2(1)_ = 0.6 ± 0.09°, and *X*_1/2(2)_ = 6.1 ± 1.1°). The distribution of half-angles shown in Figure 3*F* also indicated two basic populations of hair cells, separable either side of *X*_1/2_ = 2°, so we also calculated separate averages of the stimulus-response relations for hair cells above and below this threshold, as shown in Figure 6*B*. The slope factors and half-angles describing the transfer function of these two populations were *X*_S(<2°)_ = 0.5 ± 0.04 and *X*_1/2(<2°)_ = 0.7 ± 0.04 (red trace), and *X*_S(>2°)_ = 1.8 ± 0.3 and *X*_1/2(>2°)_ = 5.0 ± 0 (blue trace). Separating these populations according to *X*_1/2_ revealed another important functional difference: hair cells of low sensitivity were completely rectifying with a relative set point of one while cells of high sensitivity had a relative set point of 0.8.

The overall sensitivity of the neuromast was quantified as the first derivative of the stimulus-response relation (Dayan and Abbott, 2001). The thick dashed line in Figure 6*C* shows this quantity for the average output of all 67 hair cells, from which three features stand out. First, the neuromast achieves maximum sensitivity at deflections of just ∼40 nm at the tip of the hair bundle. Second, deflections from rest can be signaled with a sensitivity ∼63% of maximum, either as an increase or a decrease in glutamate release, and this is made possible by the relative set-point of the high-sensitivity population of hair cells. Third, the high-sensitivity population saturates at deflection of ∼220 nm, but the dynamic range of the neuromast as a whole is extended up to ∼1 μm by the low-sensitivity population. Acting together, these two groups of hair cells make the neuromast very sensitive to small deflections of the cupula while maintaining a large dynamic range.

### Heterogeneous adaptive properties of hair cells within individual neuromasts

The use of primary receptors of differing sensitivity is one strategy by which sensory systems maintain sensitivity over a range of stimulus strengths. A second strategy is to prevent saturation by adaptation, a change in sensitivity that often manifests itself as a time-dependent decrease in the response of a sensory neuron when a constant stimulus is applied (Adrian and Zotterman, 1926; Wark et al., 2007). In the lateral line, ramped deflections of the cupula cause the spike rates in the afferents to adapt strongly (Haehnel-Taguchi et al., 2014) but it is unclear whether this is primarily a postsynaptic effect or whether there is also a presynaptic component to adaptation.

**Figure 7.**
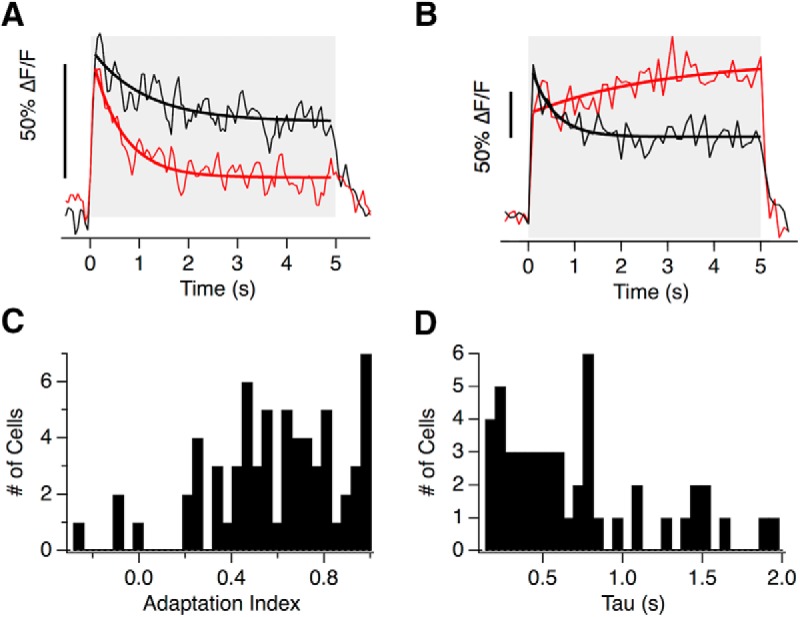
Heterogeneous adaptive properties of hair cells. ***A***, The glutamate release of two hair cells from the same neuromast adapts to different extents to a 5 s step deflection. The red one adapted with a time-constant of 0.61 ± 0.09 s (solid line) with an adaptive index of 0.65. The black hair cell adapted with a time-constant of 1.09 ± 0.32 s with an adaptive index of 0.42. ***B***, The iGluSnFR signal from two hair cells of a second neuromast. Note that while one adapted (black, AI = 0.53) the second sensitized (AI = −0.13). ***A***, ***B***, Time-constants were derived from single exponential fits, thick lines). ***C***, Distribution of adaptation index from 65 hair cells stimulated with a 5 s step. ***D***, Distribution of the decay time constants from the 55% of hair cells that could be fit with tau <2 s. In the remainder tau was >2 s and could not be estimated.

To investigate the adaptive properties of the neuromast we applied saturating or near-saturating pressure steps of 2 s or more. In 61 of 65 hair cells adaptation was apparent as synaptic depression but there was a large degree of variability within the same neuromast. For instance, Figure 7*A* shows an example in which glutamate release fell to ∼30% of peak in one hair cell (red) but only to 70% of peak in another (black). We quantified the reduction in glutamate release as an AI, which varied from 1 (complete recovery of the iGluSnFR signal to 0) through zero (no change) to negative values (reflecting an acceleration of glutamate release; detailed in Materials and Methods). The distribution of AIs measured at the end of a saturating 5 s pressure step varied widely, as shown by the distribution in Figure 7*C*. The speed of decay of the response could be described by a time-constant of 2 s or less in 74% of cells, and the distribution in this subpopulation is shown in Figure 7*D*. The remaining cells had a decay time-constant >2 s that could not be estimated reliably from a 5 s record. The shortest time constant we observed was 130 ms and 30% of all cells had a decay constant <500 ms.

Here we have used the term adaptation to mean the decline in the response to a maintained stimulus but it should be noted that this term is often used in a more specific way when characterizing the MET current providing the input to the hair cell. At this stage, adaptation can be measured as a change in the WR of the MET current that “resets” the deflection at which maximum sensitivity is achieved without significantly altering that maximum (Howard and Hudspeth, 1987; Shepherd and Corey, 1994; Holt et al., 2002; for review, see Ricci and Kachar, 2007).

These results demonstrate that hair cells of the lateral line apply different temporal filters: in some, a step stimulus generates a transient response that signals the onset of the deflection most strongly (Fig. 7*A*, red trace), whereas in others the output is more sustained and effectively signals the duration (Fig. 7*B*, black trace). An analogy can again be made with the transformation of signals in the retina, where the distinction between transient and sustained neurons has long been recognized (Baccus, 2007; Masland, 2012).

### Population signaling of a return to rest

**Figure 8.**
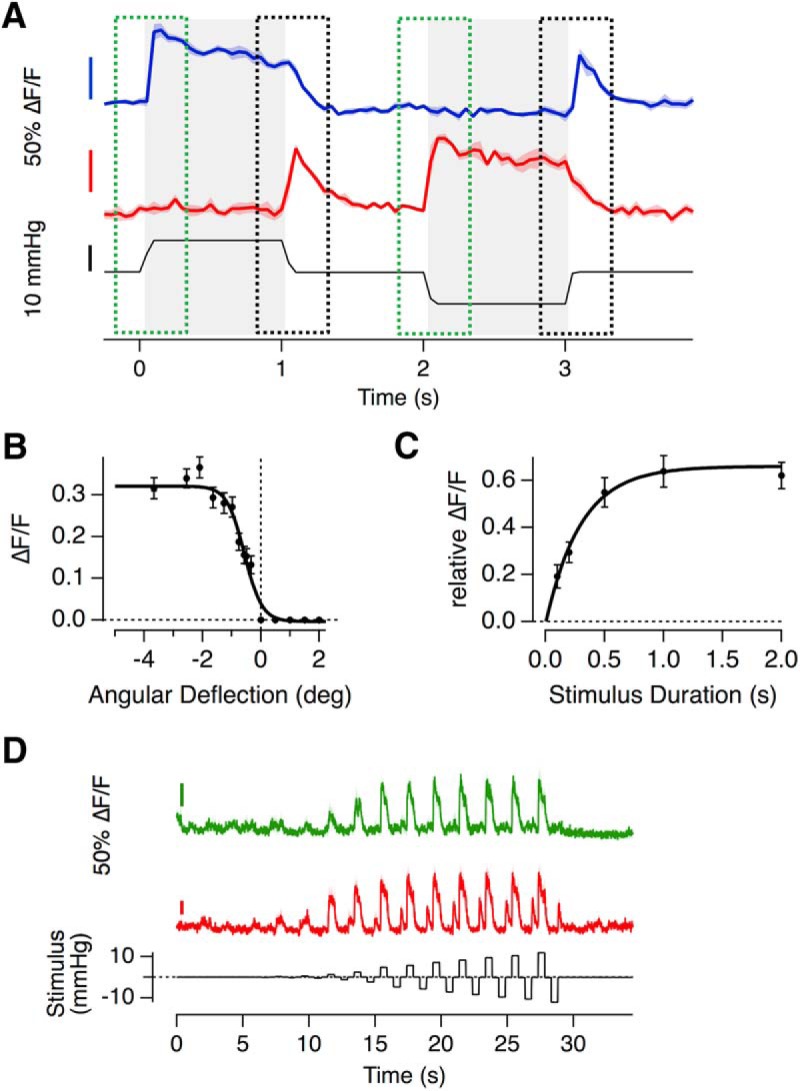
Hair cells signaling a return to rest. ***A***, Example of iGluSnFR responses of two hair cells (not from the same recording), that generate a rebound-response; a large and transient release of glutamate after the release of a deflection in the null direction. ***B***, The relationship between angular deflection in the null direction and amplitude of the rebound response. Data were averaged from 33 hair cells in which the largest rebound response exceeded 20% of the maximum response in the preferred direction (*R*_max_ = 0.32 ± 0.01, *R*_min_ = 0 ± 0.01, *X*_1/2_ = −0.56 ± 0.05 and *X*_s_ = 0.3 ± 0.05). ***C***, The relationship between duration of deflection in the null direction and the magnitude of the rebound response (as a fraction of the response to stimulation in the preferred direction). These experiments were performed with large deflections generating the maximum rebound. Results are described by an exponential that yields a time constant of 0.3 s for the development of the rebound response. ***D***, Two hair cells within the same neuromast, one of which (red) displaying a strong rebound response, whereas the other (green) does not, despite experiencing the exact same cupula deflection The relationship between cupula deflection and applied pressure in this neuromast was 0.29 deg/mmHg.

Here we have shown that push-pull signaling within a neuromast is facilitated by the high-sensitivity population of hair cells with relative set-points <1 (Figs. 5, 6*B*) and adaptation indices close to 1 (Fig. 7). Acting together, these features allow the neuromast to signal effectively the onset of a weak stimulus. A third and distinctive property of a subset of hair cells was the generation of a large glutamate transient at the offset of a stimulus. An example of this behavior is shown in Figure 8*A*: these two synapses were of opposite polarity and completely rectifying such that a deflection in the nonpreferred direction did not generate a response (green boxes). A return to rest from the nonpreferred direction did, however, generate a strong and transient release of glutamate (black boxes), equivalent to 88% of maximal response in the preferred direction. In other words, only one of the two hair cells signaled a deflection from rest, although both signaled a recovery to rest. To assess the numbers of hair cells generating such a reset or “rebound” signal we set a criterion that it must exceed 20% of the maximum response to a stimulus of the same magnitude in the preferred direction: 33 of 55 hair cells from 9 separate neuromasts generated such responses after steps of 1 s duration. Two observations ruled out the possibility that this signal was generated by an overshoot or “swingback” of the cupula at the end of the step: such a swingback was not observed when imaging the cupula at 20 Hz (Fig. 2) and measurements within the same neuromast demonstrated that whereas some hair cells generated a reset response others did not (Fig. 8*D*).

The amplitude of the reset signal depended on both the magnitude and duration of the preceding deflection in the nonpreferred direction. The dependence on magnitude could be described by a two-state Boltzmann equation with a half-angle of −0.6° (50 nm; Fig. 8*B*) and during a large deflection the response developed with a time-constant of 0.3 s (Fig. 8*C*). The reset signals transmitted through the hair cell synapse are qualitatively similar to the process of “negative adaptation” described in the MET current of a variety of hair cells and might, therefore, be driven by these channels (Holt et al., 2002; Hirono et al., 2004; Stauffer et al., 2005; Hudspeth, 2014).

## Discussion

Directly measuring the mechanical stimulus, deflection of the cupula, and the synaptic output, release of glutamate, has allowed us to specify the transfer characteristics of hair cells within neuromasts of the lateral line. We find that the input to this sensory system is determined by populations of hair cells with heterogeneous transfer characteristics (McHenry et al., 2009; Stewart et al., 2013; Olive et al., 2016; Oteiza et al., 2017). A comparison can be made with early visual processing, where the visual signal is decomposed through a variety of pathways or “channels” specialized to transmit different types of information, such as the ON and OFF channels signaling light increments or decrements, or the transient and sustained channels signaling fast or slow changes in light intensity (Baccus, 2007; Kastner and Baccus, 2011; Masland, 2012). Similarly, individual neuromasts can encode a mechanical stimulus through a number of functional channels varying in sensitivity, polarity, and temporal and adaptive properties. Here we discuss how these variations contribute to the information that can be transmitted as well as the mechanisms by which they might arise.

### Sensitivity, WR, and set-point

The high-sensitivity group of hair cells operated with half-angles ∼1.5° (Fig. 3*F*), which corresponds to a displacement of 130 nm at the tip of the stereocilia and is comparable to the sensitivity of auditory and vestibular hair cells in mice and other species (Fettiplace and Kim, 2014). This subset of hair cells saturated at deflections of ∼220 nm (Fig. 6*B*), which is much narrower than the WR of the neuromast as a whole (Haehnel-Taguchi et al., 2014). The difference can be accounted for by the second, low-sensitivity population of hair cells that extended the dynamic range of the neuromast beyond 1 μm. A strategy for detecting stimuli with a mixture of high- and low-sensitivity receptors allows the neuromast to encode weak stimuli effectively while limiting saturation.

An important feature of the high-sensitivity hair cells was a relative set-point that allowed small deflections in either direction to modulate glutamate release (Figs. 5, 6). The sensitivity to deflections from rest achieved 63% of the maximum measured at ∼40 nm (Fig. 6*C*) so it seems likely that deflections <50 nm will be transmitted to targets in the hindbrain. The behavioral significance of detecting such small deflections is, however, harder to judge and would require one to measure (or perhaps calculate) deflections of the cupula in a motile fish. One possibility is that the high-sensitivity group of hair cells are involved in the sensing of flow velocity gradients around the body of the fish that have recently been shown to underlie rheotaxis in the absence of visual input (Oteiza et al., 2017). Low-sensitivity hair cells would then be available to encode stronger stimuli triggering reflexes such as the escape response, a locomotor behavior that rapidly propels the fish away from a threat.

An important limitation of the current experiments was the sampling rate of iGluSnR signals. Imaging at 20 Hz, we were effectively blind to events occurring within the first 50 ms of an applied stimulus. The displacement generated by intermediate pressure steps settled within 50 ms (Fig. 2*H*,*I*) but ribbon synapses are capable of releasing vesicles within milliseconds of calcium current activation (Keen and Hudspeth, 2006; Li et al., 2009). It may therefore be that these experiments underestimate the peak sensitivity of hair cells assayed at their output. Improved estimates might be obtained by sampling both the iGluSnFR signal and cupula deflection at rates of 100 Hz or more, although this is unlikely to be worthwhile until the signal-to-noise ratio of these imaging methods are improved significantly.

### Possible mechanisms underlying variations in sensitivity and set-point

The input/output relation of the hair cell is determined by a chain of events, several of which might be sources of variation. The first step to consider is the mechanical coupling between the cupula and the hair bundles embedded within it. It is not known whether the hair bundle is fixed within the cupula to follow it exactly or whether relative displacements might occur large enough to modulate glutamate release. If the set position of different hair bundles varies because of interactions with the cupula then the resting transducer current and the operating set-point could be different. Similarly, if the attachment points of the kinocilia within the cupula or the stereocilia to the cupula varied this would alter the mechanical stimulus applied to different hair bundles, which would manifest itself as variations in the sensitivity of different hair cells. Variations in the relationship between hair bundle displacement and the MET current have also been observed within a single experiment in the auditory and vestibular systems (Holt et al., 1997; Stauffer and Holt, 2007).

A change in sensitivity measured at the output might also reflect the process of Ca^2+^-triggered exocytosis at the active zone. It has been shown, for instance, that the amplitude of calcium signals varies between hair cells within a neuromast of the larval zebrafish, reflecting different levels of developmental maturity (Kindt et al., 2012, Olt et al., 2014). It may therefore be that the less-sensitive population of hair cells that we have identified by their transfer function is synonymous with hair cells that are functionally immature with smaller calcium currents. A heterogeneous population of hair cells at different levels of maturity is likely to continue into adulthood because hair cells in neuromasts turnover continuously as they are damaged and regenerate (Cruz et al., 2015). Even within a single hair cell active zones may vary in the number of calcium channels and their voltage dependence, leading to different release rates driven by the same receptor potential (Ohn et al., 2016). Finally, variations in sensitivity at the output might also reflect differences in the efficiency with which a given Ca^2+^ signal triggers exocytosis (Olt et al., 2014).

The set-point for signaling the output from a hair cell will depend on where the resting potential sits relative to the threshold for activation of calcium channels. Push-pull modulation of glutamate within individual hair cells requires that the resting potential be in a range where Ca_V_1.3 channels under the ribbon are activated sufficiently to drive vesicle fusion (Platzer et al., 2000; Olt et al., 2014). If the resting potential is hyperpolarized relative to the threshold the output of the hair cell will be completely rectifying, as observed in the low-sensitivity population of hair cells. Conductances that might cause resting potentials to vary include K^+^ channels in the basolateral membrane (Olt et al., 2014) and *I*_h_ inward rectifiers (Trapani and Nicolson, 2011). The voltage dependence of Ca_V_1.3 channels may also be subject to modulation that alters the threshold for activation and the steepness of the current–voltage relation (Striessnig et al., 2010).

### Adaptation

We also found large variations in the degree and speed of adaptation between different hair cells within a neuromast (Fig. 7). Such a mixture of responses will help the population within a neuromast to signal both sustained stimuli, such as water flow (Voigt et al., 2000), as well as sudden deviations, such as eddy currents or the motion of other creatures in their immediate environment (McHenry et al., 2009). There are likely to be a number of processes that contribute to adaptation in the synaptic output measured using iGluSnFR, beginning with, but not confined to, adaptation at the MET channel. In hair cells of the auditory and vestibular systems a second major cause of adaptation measured at the output is vesicle depletion leading to depression at the ribbon synapse (Schnee et al., 2005, 2011; Goutman, 2017). These two processes have been studied in isolation, but the present study has assessed how they act together to adjust the input/output relation of the hair cell.

The kinetics of adaptation of the MET current can generally be described by two time constants, τ_fast_ in the milliseconds range and τ_slow_ in the tens of milliseconds range (Shepherd and Corey, 1994; Holt et al., 1997; Vollrath and Eatock, 2003; Stauffer and Holt, 2007). It is not, however, clear whether these processes are mirrored in the exocytic response of the hair cell. Ribbon synapses contain a pool of docked vesicles that can be released completely within ∼20 ms when the calcium current is activated strongly (Burrone and Lagnado, 2000; Beutner et al., 2001), but such a fast and transient response would not have been resolved in the present experiments. We were, however, able to resolve adaptation of the output that occurred on time-scales of hundreds of milliseconds to seconds (Fig. 7). These relatively slow kinetics indicate that the rate-limiting process was downstream of the MET channel and a strong possibility is that adaptation reflected depletion of releasable vesicles at the active zone. Indeed, capacitance measurements of exocytosis in hair cells of the lateral line demonstrate that a strong step depolarization stimulates release that decays with time constants of ∼500 ms (Eatock, 2000; Lv et al., 2016), whereas optical measurements at ribbon synapses in the retina demonstrate that these can also depress with time-constants of a few seconds (Nikolaev et al., 2013).

A related property was so-called negative adaptation, whereby deflections of the hair bundle away from the kinocilium primed the hair cell to generate a transient burst of glutamate release when the cupula returned toward rest (Fig. 8). This response signaled the offset of a stimulus and was a function of both the amplitude and duration of the preceding deflection, thereby encoding the integrated stimulus. Negative adaptation within the neuromast generates a population signal that encodes the cessation of a stimulus, as would occur, for instance, in the intervals between swimming bouts (Russell and Roberts, 1974; Palmer et al., 2005). The growth of the reset signal with time and angle of deflection indicate that it has the potential to be used to integrate flow gradients along the body of the side of the fish, the detection of which underlies rheotaxis (Oteiza et al., 2017).
